# Evaluating e-health literacy, knowledge, attitude and practice regarding COVID-19 prevention and Self-Protection among Iranian students: a cross-sectional online survey

**DOI:** 10.1186/s12909-022-03210-3

**Published:** 2022-03-05

**Authors:** Sareh Dashti, Dina Abadibavil, Nasibeh Roozbeh

**Affiliations:** 1 Department of Midwifery, Faculty of Nursing and Midwifery, Mashhad Medical Sciences, Islamic Azad University, Mashhad, Iran; 2 Mother and Child Welfare Research Center, Hormozgan University of Medical Sciences, Bandar Abbas, Iran

**Keywords:** E-health literacy, Coronavirus 19, Knowledge, Attitude, Practice, Preventive behavior, COVID19

## Abstract

**Background:**

The coronavirus 2019 (COVID-19) pandemic requires integrated intervention by both the governments and individuals. University students have a great role in distributing reliable information about disease prevention behaviors. The aim of this study was to identify the Knowledge, Attitude and Practice of COVID-19 Prevention and Self-Protection behaviors in students.

**Methods:**

This cross-sectional online survey was conducted on Iranian university students. All students filled a questionnaire consisting of demographic characteristics, e-Health Literacy Scale (EHEALS) questionnaire and a researcher-made knowledge, attitude and practice (KAP) questionnaire. Data was analyzed using the SPSS software.

**Results:**

A total of 925 students (69.9% female) participated in this study. The median age of the students was 23 years old. Majority of students (641, 69.3%) were non-medical students. The median and interquartile range (IQR) for knowledge, attitude, practice and P-EHEALS scores in Medical students were 52.00 (27.00), 4.00 (1.00), 28.00 (8.00), and 26.00 (9.00), respectively which were significantly higher than non-medical students, 28.00 (15.00), 3.00 (2.00), 20.00 (8.00), and 26.00 (9.00), respectively (*p* < 0.001). Practice score was a significant related to knowledge (*p* < 0.001), attitude (*p* < 0.001), having a COVID-19 infected family member (*p* < 0.001), older age (*p* < 0.001), medical field of education (*p* = 0.001), higher EHEALS score (*p* = 0.018), and female gender (*p* = 0.013). Knowledge, attitude and having a COVID-19 infected person in family were the strongest predictors of preventive practices.

**Conclusions:**

KAP and E-Health literacy of university students, especially non-medical students, should be considered in order to improve COVID-19 preventive behaviors in the society.

## Background

The emergence of infectious viral pandemics is a great challenge in the Twenty-First century [1]. Coronaviruses belong to a large family of viruses that cause a large spectrum of respiratory diseases ranging from common cold to more severe conditions including severe acute respiratory distress syndrome (SARS), Middle-East respiratory distress syndrome (MERS) and coronavirus 2019 (COVID-19) [2]. COVID-19 is a new virus that had a negative effect on the lives of people globally and has caused serious concerns [3–5]. Based on the report by the World Health Organization (WHO), COVID-19 started in Wuhan, China in December 2019 and infected a large number of people worldwide [6]. In January 2020, WHO declared COVID-19 a global concern that requires an integrated global reaction [7]. As the virus spread is airborne, the virus spread in almost all the countries within one month [8]. The virus primarily spreads through close contact especially through cough and sneeze that spread droplets as far as 6 m [8, 9]. The Center for Disease Prevention and Control (CDC) reported that the incubation period of COVID-19 is approximately two weeks [8]. As symptomatic patients were quarantined, the main source of virus spread were asymptomatic patients and individuals in their incubation period [10]. Therefore, it became mandatory for all the population to follow social distancing [10]. To date, no definite treatment has been found for COVID-19 [8]. Therefore, due to the contagious nature of the disease and lack of quarantine infrastructure as well as lack of a definite treatment, tele-health and telemedicine have been used by countries in performing health care services in the COVID-19 pandemic era [11–13].

Governments are responsible for promoting the knowledge and providing the required infrastructures for social distancing and COVID-19 prevention. People’s knowledge on these issues is a detrimental factor in the success in disease prevention and control [14]. Internet is a means for health education. The advantages of using internet in education include low costs, speed in finding the answers to questions, accessibility and anonymity [10, 15]. Therefore, e-health and e-health literacy were introduced as a new entity in the field of health education. E-health is defined as the use of information technology for improvement of in health and healthcare services [16]. E-health literacy is defined as the ability to search, find, review and evaluate health information from electronic sources. Internet use, including opportunities, possible harms, and equality in public access to internet, literacy level, age and gender, other than the conventional factors affect health literacy. [16]. Currently various online health education sources are providing education materials. These sources can be categorized into special and non-special sources. The special sources provide health information by professionals and include governmental, at ministerial, medical school or medical faculty levels, private websites, personal weblogs, websites of health and medical clinics and private organizations. The non-specific sources provide information from unprofessional informants that mainly pertain to health-related news, medical and health related information for public users, information regarding medical and healthcare clinics, doctors and hospitals, as well as consulting users in the choice of services. E-health was shown to have a significant effect on knowledge, healthy behavior and ability in following up medical services [17]. E-health has a crucial role in healthcare decision making and disease prevention behaviors, including hand hygiene, wearing masks and other preventive strategies [18]. Low level of e-health literacy is associated with poor understanding of health information and medical recommendations for disease prevention. Therefore, low e-health level can lead to reduced use of preventive services, regular physician visits, increased duration of admission and increased treatment costs and subsequently increased morbidity and mortality [19]. Iran is one of the countries affected by COVID-19 [20]. Among all people, it is essential to pay attention to students, especially medical students, because of their place in society and having basic information about health issues, traveling in high-risk environments and dealing with COVID-19 patients [21]. It seems that lack of knowledge regarding the routes of transmission and preventive rules for COVID-19 and awareness about the risks of COVID-19 infection can significantly influence the success in COVID-19 prevention strategies. University students comprise the educated portion of the society and are supposed to have high level of knowledge, attitude and practice towards health and care issues. Their knowledge, attitude and practice not only affect their own lives but also has an influence on the society. On the other hand, the level of knowledge of university students on health issues can be a determinant for the efficiency of education provided by the authorities. To the best of our knowledge no study has yet assessed the e-health literacy about COVID-19 in Iran or in the world. Furthermore, no study has yet been conducted in Iran regarding the relationship between e-health literacy and knowledge, attitude and practice (KAP) of students regarding disease prevention behaviors. Understanding the relationship between KAP and e-health can help stakeholders in planning preventive strategies in order to design effective interventions in the shortest possible time. Therefore, the aim of this study was to identify the Knowledge, Attitude and Practice of COVID-19 Prevention and Self-Protection behaviors in students.

## Methods

### Subjects and design

This cross-sectional study was conducted on medical and non-medical university students in Iran based on online survey. The calculated sample size for this study was 925 subjects. Subjects were recruited based on convenience sampling.

Inclusion criteria were being Iranian citizen, studying in a university in Iran, and willingness to participate in the study. Exclusion criteria were incomplete questionnaires, being a fresh graduate and Iranian citizen who were studying in foreign universities.

### Study procedure

An Online questionnaire was designed and prepared by researchers and was made available through a link. The link was presented in social media groups that included the highest number of medical and non-medical students.

Students were notified about the online questionnaire through a link provided via social media groups. In the first page of the survey, the purpose of the study was explained to the students and students could only access the questions if they agreed to participate in the study by clicking on I agree bottom. Subjects could only fill the questionnaire once.

### Study instruments

The online questionnaire consisted of demographic, KAP and e-health literacy questionnaires. Demographic questionnaire consisted of characteristics including age, sex, marital status, type of university, and level of education as well as history of COVID-19 infected in a family member, duration of daily internet use, being vaccinated for influenza in 2019, and possessing thermometer at home.

The KAP questionnaire was a researcher-made instrument that assessed the level of knowledge, attitude, and practice of the students regarding symptoms, progression, prevention and treatment of COVID-19. The KAP questionnaire was prepared in Persian language and included 40 questions that assessed knowledge (scores ranging from 0 to 83), four questions that assessed attitude (scores ranging from 0 to 4) and 40 questions that assessed practice (scores ranging from 0 to 40) (Table 1). The face and content validity of the questionnaire was assessed by an expert panel consisting of a PhD graduate in Reproductive Health with an experience in design and validating questionnaires and a Medical doctor. The reliability of the questionnaire was assessed on a group of 55 students, who were not entered the main study. The overall Cronbach’s alpha for the questionnaire was 0.778.

**Table 1:** Content of the KAP and e-Health literacy questionnaires

Questionnaire	Item
e-Health literacy	I know how to find valuable information sources in the internet
	I know how to use internet to answer my health questions
	I know the health information sources in the internet
	I know where to find useful health information in the internet
	I know how to use online information to meet my health needs
	I have the required skills to evaluate online health information
	I can categories online health information sources from very useful to very useless
	I have high self-confidence in using internet to meet my health and wellness needs
	I prefer to use Persian language in searching health websites
	I have the required self-confidence in searching health information online
	On average, I pass …. hours using internet
	Health condition (healthy, COVID-19 infected, healthy but having a friend or family member with the disease and looking for health information)
	I use general/specific websites to obtain health related information
Knowledge	Symptoms of COVID-19
	Alarming symptoms in COVID-19
	High risk population for acquiring COVID-19
	Percentage of infected people who develop symptoms
	Percentage of infected people who are hospitalized
	Percentage of infected people who die due to COVID-19
	The mean number of people an infected person can infect
	High risk places for becoming infected
	The cause of COVID-19
	Is the cause of COVID-19 influenza virus?
	Coronavirus is not a new virus and previous incidents of coronavirus infections existed in the past
	Symptoms of all cases of COVID-19 is like common cold
	Coronavirus is transmitted through respiratory droplets
	Traveling with a COVID-19 carrier causes infection
	Coronavirus is transmitted through insects
	The coronavirus family can infect both humans and animals
	Children do not develop COVID-19
	Hot bath can destroy the virus in the body
	Heating mouth and nose with hair drier can destroy the virus in the respiratory tract
	Sauna can destroy the virus in the body
	Hot vaporizers can destroy virus in the respiratory tract
	Hand driers cannot destroy the virus alone
	Rinsing nose with normal saline can protect against COVID-19
	Using mouthwash can protect against COVID-19
	Drinking alcohol can protect against COVID-19
	Garlic can protect against COVID-19
	Washing hands with alcohol sanitizers is more effective compared to hand washing with water and soap
	Asymptomatic patient cannot be a carrier
	Hand washing with plain water is not enough to eliminate the virus
	There is no preventive medication for COVID-19
	Influenza vaccination cannot protect against COVID-19
	There is no definite medication for COVID-19
	Those who pass course of COVID-19 are immune to this virus for the rest of their lives
	All infected patients need to be hospitalized
	COVID-19 always produces mild symptoms
	Only febrile COVID-19 patients can transfer the disease to others
	Those who pass course of COVID-19 do not need to follow hygiene tips and social distancing rules
	Infected patients and those who were in contact with them should be quarantined for 14 days
	Do you think you have sufficient information about COVID-19?
	Sources of obtaining information regarding COVID-19
	Do you need more information about COVID-19?
Attitudes	Health related suggestion to COVID-19 patients
	Perception about the health threats of COVID-19
	Prediction of the results of global response to COVID-19
	Estimation of the duration to contain COVID-19
Practice	Visiting infected relatives
	Policy makers need to focus on more important issues than COVID-19
	All infected cities should be immediately quarantined
	Social distancing and healthy recommendations should be strictly followed
	Anyone with a history of travel to contaminated areas or close contact with infected patient should inform the Ministry of Health
	I prefer to by online instead of going shopping
	If I could not by a mask, I will make one at home
	Only high-risk groups should follow the recommendations
	COVID-19 pandemic will end with the beginning of hot season
	Everybody should use his/her personal towel or disposable tissue at home
	Pet owners should watch contacts between their pets and themselves and others
	Relatives of deceased people should not feel shame
	Although I follow social distancing recommendations, I do not have a good feeling about visiting cured patients
	Lockdown made the opportunity for me to go and visit my relatives and family members
	Do you have a COVID-19 patient in your house?
	What measures you perform if you have a COVID-19 patient at home?
	What do you do to reduce stress due to the condition?
	Have you infected with the Coronavirus?
	What measures you perform if you have a COVID-19?
	Whish of the social distancing recommendations did you follow?
	How many hours you wear mask?
	What type of mask have you used during the COVID-19 pandemic?
	How many times did you leave home in the past week?
	Why did you leave home in the past week?
	How many times do you wash your hands every day?
	How many seconds does your hand washing last?
	How many times do you use hand sanitizers a day?
	How many seconds do you rub your hands with hand sanitizers?
	How many times did you leave your city/town from the initiation of the pandemic?
	Why did you leave your city/town from the initiation of the pandemic
	Have you lost family member(s) or friend(s) due to COVID-19
	I have prepared medications to counter COVID-19 at home
	I have purchased masks to protect myself
	I have made masks for myself and my family at home
	I carry hand sanitizer or soap with me when I am outside my home
	I participated in funerals during the COVID-19 pandemic
	I weekly go to graveyard
	I went shopping in the final days of the year (before the Persian New Year)
	I wash groceries with water and soap when I bring them home
	I heat bread before consumption to kill the virus on its surface
	I drank alcoholic drinks to combat the virus
	I let my children play outside during the lockdown
	I let my children visit their gran parents
	I frequently sanitize touched surfaces (TV remote control, door handles, table surface, computer keyboard and mouse)
	I do not use my cell phone outside house to prevent it from contamination
	I often sanitize my cell phone based on the recommendations
	I participated in new year family gatherings similar to previous years
	I will quarantine myself at home if I have symptoms like fever, cough, or shortness of breath
	I started hoe quarantine after the authorities passed the motion restriction act
	I use mask if I had to leave home
	I sanitize my hands with alcohol or wash them before touching my face
	I cover my mouth and nose before cough and sneeze
	I did not travel during the new year holiday

E-Health literacy of the students was assessed using the Persian translate of the e-Health Literacy Scale (P-EHEALS) questionnaire. This questionnaire consisted of 8 questions that were scored based on a 5-point Likert scale (Table 1). The reliability and validity of the P-EHEALS questionnaire was previously assessed in a study on Persian students (overall Cronbach’s alpha was 0.89) [22].

### Statistical analysis

Statistical analysis was conducted using the statistical package for social sciences (SPSS) version 16. The normality of continuous data was assessed using the Kolmogorov–Smirnov test. As data was not normally distributed median and interquartile range (IQR) were used to present data. Frequency and percentage were used to present categorical data. Comparison of data between medical and non-medical groups was performed using the Mann–Whitney test. Comparison of the distribution pattern of categorical data between groups was conducted using the chi-square test. In order to assess the relationship between theory compartments and outcome, a regression model was created with practice score as target and other study variables as predictors. Level of statistical significance was *p* < 0.05.

## Results

Of the total 6100 target population 925 completed the online questionnaire (15% response rate). The median and IQR for age of the students were 23.00 (4.00) years. Majority of students (647, 69.9%) were female. Of the 925 students 641 (69.3%) were studying in non-medical fields, while 284 (30.7%) were studying in medical fields. Demographic characteristics of the students are shown in Table 1. There was a significant difference in gender distribution (*p* < 0.001), level of education (*p* < 0.001), type of study (*p* = 0.006), academic semester (*p* < 0.001), marital status (*p* = 0.011), history of COVID-19 infection in family (*p* < 0.001), and influenza vaccination in the previous year (*p* < 0.001) between medical and non-medical students (Table 2).

**Table 2:** Demographic characteristics of the students as per education groups

Variable		Total *N* = 925	Medical *n* = 284	Non-medical *n* = 641	*P*
Age (years)		23.00 (4.00)	22.00 (3.00)	23.00 (4.00)	0.119 Ɨ
Gender	Male	647 (69.9%)	244 (85.9%)	403 (62.9%)	< 0.001*
	Female	278 (30.1%)	40 (14.1%)	238 (37.1%)	
Level of education	Diploma	133 (14.4%)	25 (8.8%)	108 (16.8%)	< 0.001*
	Bachelor	599 (64.8%)	209 (73.6%)	390 (60.8%)	
	Masters	152 (16.4%)	28 (9.9%)	124 (19.3%)	
	PhD	41 (4.4%)	22 (7.7%)	19 (3.0%)	
Type of university	Semi-governmental (Azad)	348 (37.6%)	130 (45.8%)	218 (34.0%)	0.006*
	Public	543 (58.7%)	147 (51.8%)	396 (61.8%)	
	Private	21 (2.3%)	5 (1.8%)	16 (2.5%)	
	Part time (Payam Noor)	13 (1.4%)	2 (0.7%)	11 (1.7%)	
Academic semester		4.00 (3.00)	5.00 (3.00)	3.00 (3.00)	< 0.001 Ɨ *
Marital status	Single	737 (79.7%)	212 (74.6%0	525 (81.9%)	0.011*
	Married	188 (20.3%)	72 (25.4%)	116 (18.1%)	
Duration of internet use (hours/day)		5.00 (4.00)	6.00 (4.00)	6.00 (4.00)	0.109 Ɨ
History of COVID-19 infection in family		524 (56.6%)	110 (38.7%)	414 (64.6%)	< 0.001*
Influenza vaccination last year		394 (42.6%)	88 (31.0%)	306 (47.7%)	< 0.001*
Having thermometer at home		520 (56.2%)	173 (60.9%)	347 (54.1%)	0.055

*PhD* Doctor of Phylosophy

Ɨ Median and interquartile range were used and the comparison was conducted using Mann–Whitney test

Frequency and percentage were used for all data except for age and the comparison was conducted using chi-square test

The scores of the knowledge, attitude, practice and EHEALS questionnaires are shown in Table 2. There was a significant difference in terms of knowledge, attitude, practice and EHEALS scores between medical and non-medical students (p < 0.001 each) (Table 3).

**Table 3:** Knowledge, attitude, practice and EHEALS scores of the students as per education groups

Variable	Total *N* = 925	Medical *n* = 284	Non-medical *n* = 641	p
Knowledge score	32.00 (27.00)	52.00 (27.00)	28.00 (15.00)	< 0.001*
Attitude score	3.00 (2.00)	4.00 (1.00)	3.00 (2.00)	< 0.001*
Practice score	22.00 (11.00)	28.00 (8.00)	20.00 (8.00)	< 0.001*
P-EHEALS score	28.00 (9.00)	30.00 (8.00)	26.00 (9.00)	< 0.001*

Median and interquartile range were used and the comparison was conducted using Mann–Whitney test

The best fitted linear regression model to identify the predictors for practice score as the outcome is shown in Table 3. The overall R squared vale of the model was 0.655 indicating the 65.5% of the variance in practice score could be explained by the model. Based on the regression model there was a significant relationship between practice score and knowledge score (*p* < 0.001), attitude score (*p* < 0.001), having a COVID-19 infected person in family (*p* < 0.001), age (*p* < 0.001), medical field of education (*p* = 0.001), EHEALS score (*p* = 0.018), and female gender (*p* = 0.013) (Table 4). The most powerful predictor of practice score was knowledge, followed by attitude, having a COVID-19 infected person in family and age.

**Table 4:** Relationship between practice score and other variables

Variable	Standardized Beta	p
Knowledge score	0.516	< 0.001*
Attitude score	0.227	< 0.001*
COVID-19 infected person in family	0.113	< 0.001*
Age	0.109	< 0.001*
University type	-0.027	0.170
Non-medical field of education	-0.081	0.001*
EHEALS score	0.049	0.018*
Male gender	-0.050	0.013*

^*^ Significant relationship

## Discussion

Health literacy is a central issue in controlling the global spread of COVID-19 through disease prevention and preparation of health systems for combating the disease in a short period of time [23]. The WHO indicated that low level of health literacy has a role in global health issues. For instance, nearly half of the European adult population had unacceptable health literacy and were not eligible to care for themselves or others [24]. University students, especially medical students, have an important role in preventing COVID-19 spread, obtaining correct information from credible online sources. The aim of this study was to assess the relationship between COVId-19 prevention knowledge, attitude, and practice, e-health literacy and demographic and academic parameters among university students.

The findings of this study showed that the level of knowledge and attitude of medical students about COVID-19 were acceptable (above 70% of the maximum score). However, the level of knowledge in non-medical students. Practice score was low in both the medical and non-medical students. Medical students had higher scores in all KAP fields. Furthermore, this study showed that COVID-19 prevention practice was significantly related to knowledge, attitude, e-health literacy, having a COVID-19 infected family member, older age, studying in medical field, and female gender. Knowledge, attitude and having a COVID-19 infected family member were the most important predictors of practice followed by age, field of education, gender, and EHEALS score.

Similar to the findings of this study, a study regarding preventive behavior against influenza in among Iranian school students 2010 showed a significant relationship between practice scores and the scores of knowledge, perceived benefits, sensitivity and perceived severity [25]. The study also reported that the level of knowledge of the students was low [25]. In another study on the KAP of medical students about hospital infections, the level of knowledge and attitude were reported to be acceptable. This finding was similar to the findings of our study [26]. In a cross-sectional study on 872 university students in China, the level of knowledge regarding COVID-19 was reported to be acceptable [27]. This finding was attributed to great education campaigns that were mostly conducted through internet [27]. Similar studies indicated acceptable level of knowledge regarding COVID-19 in university students [28, 29]. University students are supposed to have high level of knowledge due to their access to academic resources and their knowledge about obtaining information from credible sources.

E-health literacy has an important role in COVID-19 prevention and control due to the emergence of the disease and social distancing rules. These conditions prevent individuals from obtaining information from face-to-face interaction with health care providers. The findings of our study showed that students with higher e-health literacy had higher knowledge and practice in preventing COVID-19. It was previously shown that E-health literacy was significantly correlated with knowledge, and improvement in health behavior and quality of life. This finding was in line with the findings of our study [30, 31].

The findings of this study showed a significant difference in level of education, type of university, academic semester, marital status, having a COVID-19 infected family member, and receiving influenza vaccine in the past year between medical and non-medical students. These findings were in line with the findings of previous studies in South Korea, Hong Kong and the United kingdom [32, 33]. A study on KAP of COVID-19 in Iranian university students showed that the level of knowledge of university students was high [28]. The study also reported that the level of knowledge was related to the field of education, marital status, academic year, number of family members and the academic performance of the students [28]. This finding was also in line with the findings of our study. Other studies that were conducted on university students in Iran reported that medical students had higher level of knowledge on COVID-19 compared to non-medical students [29, 34]. Due to the medical background and compatibility of education credits, medical students have a higher e-Health literacy and performance compared to non-medical students. As medical students have to attend clinical wards, they have higher attitude toward learning protection methods against COVID-19 to protect themselves. This might be a reason for a higher level of knowledge and attitude in medical students compared to non-medical students.

The findings of this study showed that gender was significantly related to COVID-19 prevention practices. Similarly, a previous study showed that health related behaviors, including participation in screening and prevention strategies, were more common among women compared to men. It was stated that gender has a role in perceived responsibility and ability to obtain information as well as understanding the thread due to the disease and gender preference in the society [35]. Similarly, in another study, men were found to have lower level of knowledge and practice in relation to COVID-19 prevention compared to women [36]. The findings of this study also showed that having a COVID-19 infected family member was among the predictors for COVID-19 preventive practices. Having an infected family member is an alarming sign for all family members and makes them keener to obtain knowledge and perform preventive strategies.

Furthermore, older students were found to have higher practice scores compared to younger students in our study. This finding can be justified by a higher level of knowledge due to the higher duration of academic studies and better perception of the disease among older students compared to younger students. Due to the special role of students in COVID-19 pandemic based on the findings of some studies regarding the association between higher e-Health literacy and higher general health in Iranian students [21, 37], It seems that promoting e-Health literacy regarding COVID-19 can help prevent this disease.

## Conclusion

Regarding the role of university students in preventing COVID-19 spread, there is a need to educate and enable students obtain health information from reliable online sources. Educations can be conducted through workshops to improve attitudes and motivate students in performing preventive behaviors. The findings of our study indicated that stakeholders should focus more on improving the e-Health literacy of students in order to increase their knowledge, attitude and practice regarding COVID-19. This study also indicated that non-medical students need to be educated on e-Health literacy.

## Data Availability

The collected data in this research is owned by the university and the data will be available through contact with the university through the corresponding author.
